# Systemic inflammation impairs recovery from hookworm-associated anemia in a wild marine mammal host

**DOI:** 10.3389/fimmu.2025.1659969

**Published:** 2025-11-11

**Authors:** Violetta Zaitseva, Nanami Arakawa, Carmon Co, Aranza Gomez-Camus, Diego Perez-Venegas, Felipe Montalva, Josefina Gutiérrez, Claudia Ulloa-Contreras, Ricardo Chihuailaf, Claudio Verdugo, Dorothee Bienzle, Mauricio Seguel

**Affiliations:** 1 Department of Pathobiology, Ontario Veterinary College, University of Guelph, Guelph, ON, Canada; 2 Department of Pathobiology, School of Veterinary Medicine, St George’s University, St George’s, Grenada; 3 Facultad de Ciencias de la Vida, Universidad Andrés Bello, Santiago, Chile; 4 Guafo Science Research Group, Punta Weather, Isla Guafo s/n, Quellón, Región de los Lagos, Chile; 5 Instituto de Patología Animal, Facultad de Ciencias Veterinarias, Universidad Austral de Chile, Valdivia, Chile; 6 Programa de Doctorado en Ciencias mención Ecología y Evolución, Facultad de Ciencias, Universidad Austral de Chile, Valdivia, Chile; 7 Millennium Institute Biodiversity of Antarctic and Subantarctic Ecosystems (BASE), Santiago, Chile; 8 Instituto de Ciencias Clínicas Veterinarias, Facultad de Ciencias Veterinarias, Universidad Austral de Chile, Valdivia, Chile

**Keywords:** helminth, hookworm, IL-6, IL-10, iron, otariid, neutrophil, uncinaria

## Abstract

Inflammation is a critical defense against pathogens but can impair iron metabolism and erythropoiesis, potentially causing or exacerbating anemia during infection. However, the ecological and evolutionary relevance of this trade-off remains poorly understood. Naturally co-evolved host–parasite systems offer a unique opportunity to explore how inflammatory responses balance the benefits of pathogen control against potential physiological costs. We examined how systemic inflammation affects recovery from hookworm-associated anemia in South American fur seal (*Arctocephalus australis*) pups, aiming to determine whether inflammation facilitates recovery or imposes hematological constraints. We longitudinally monitored 83 pups over approximately 3 months on Guafo Island, Northern Chilean Patagonia, measuring hookworm burden, hematological parameters, iron concentration, and blood cytokines. Seventy-two percent of the pups developed clinical hookworm infection, and 47% of these became anemic. Among anemic pups, 54% recovered from anemia 2 months after infection. Changes in inflammatory markers, but not hookworm burden, iron concentration, or body condition, predicted recovery outcome. Sustained increases in IFN-γ and neutrophils reduced the likelihood of recovery, while increased IL-10 concentration favored recovery. These effects were independent of plasma iron concentration, although IL-6 was negatively correlated with lower plasma iron. Our findings show that prolonged systemic inflammation impairs recovery from anemia in a wild marine mammal, highlighting a physiological cost of inflammation in early life as a key ecological trade-off between immune defense and hematological resilience in natural host–parasite systems.

## 1 Introduction

Inflammation is a critical component of the immune response to infection, but its activation during early life can impose substantial physiological costs. In neonatal mammals—where iron reserves are low and erythropoiesis is rapidly developing—inflammatory responses can alter iron metabolism and impair red blood cell production, exacerbating or even causing anemia (1–5). Understanding when and why a protective response essential for neonatal survival becomes harmful remains uncertain. Ecological trade-off theory suggests that restricting blood-derived resources may offer protective benefits under certain conditions (6, 7). For instance, by sustaining anemia, the host may reduce erythrocyte availability and limit resources for hematophagous parasites such as hookworms (6). However, experimental evidence often contradicts this hypothesis, showing that inflammation can promote parasite clearance and avoid hookworm-induced damage, including anemia (8, 9). These findings, however, typically arise from artificial host–parasite systems that lack a shared evolutionary history. In contrast, in naturally co-evolved host–parasite relationships—such as those found in wild populations—inflammation triggered by hematophagous parasites may serve to regulate blood resources (6, 10), potentially initiating or sustaining anemia as part of a complex adaptive response.

Wild marine mammals provide a unique opportunity to investigate host–parasite dynamics and anemia in a natural setting. For these diving mammals, adequate oxygen-carrying capacity is a critical physiological trait. Although they have physiological adaptations for diving—such as increased blood volume and elevated myoglobin stores—the basic mechanisms of oxygen uptake and delivery remain fundamentally similar to those of terrestrial mammals (11, 12). Because anemia is synonymous with reduced hemoglobin concentration and thereby impaired oxygen transport, it can have adverse effects on the survival, growth, and development of young marine mammals (13, 14). Consequently, parasites or pathogens that preferentially exploit blood resources in these hosts would likely need to be rapidly controlled or cleared to avoid substantial fitness costs (15). Such is the case for marine mammal hookworms (*Uncinaria* spp.), hematophagous nematodes that infect nearly all fur seal and sea lion (otariid) species (16). As in terrestrial mammals, hookworms of marine mammals cause anemia, growth retardation, and even mortality in young individuals (16). However, otariid pups are capable of rapidly clearing hookworm infections, with some otariid species eliminating adult parasites from the gut within 4 to 8 weeks post-infection (17, 18). This clearance is partially mediated by parasite-specific antibodies that are thought to interfere with the parasite’s ability to digest host blood (19). These findings suggest that impairing parasite access to host resources may be a key strategy for managing infection. Whether this defense occurs at the cost of restricting the host’s own access to blood resources—thereby contributing to or sustaining anemia—remains unknown.

Among marine mammals, South American fur seal (*Arctocephalus australis*) pups represent a particularly compelling model. These pups are born synchronously in large rookeries and undergo predictable postnatal exposure to hookworms (*Uncinaria pacificum*), which are transmitted through colostrum within hours of birth (20). Although nearly all pups are exposed to the parasite, clinical outcomes vary. Some pups develop severe anemia, while others recover or remain with mild clinical signs despite considerable parasite burdens (20). This natural variation offers a unique window into the interplay between parasitic infection, inflammation, and anemia during early life.

In this study, we used longitudinal data from two consecutive breeding seasons on Guafo Island, Northern Chilean Patagonia, to examine the role of inflammation in shaping the course of hookworm-associated anemia in South American fur seal pups. We integrated repeated measurements of hemoglobin, plasma iron, parasite burden, and inflammatory cytokines to investigate how systemic inflammation influences the onset and resolution of anemia. Our findings provide new insights into the immunopathological trade-offs that govern early-life health outcomes in a naturally co-evolved host–parasite system, advancing our understanding of when inflammation shifts from beneficial to detrimental.

## 2 Materials and methods

### 2.1 Animals and sample collection

We conducted this study at Guafo Island, Northern Chilean Patagonia (43°35′34.9″S, 74°42′48.53″W), during the 2022 (December 15, 2021 – March 7, 2022) and 2023 (December 14, 2022 – March 3, 2023) South American fur seal reproductive seasons;. We collected all samples from a single breeding group within the colony. We initially captured 124 pups by hand within 1 to 4 days after birth and marked them with commercial hair dye on the dorsal fur. We then recaptured them approximately every 23 days (mean ± SD = 23.0 ± 16.6 days) for up to 12 weeks. For this study, we selected pups with at least 3 recaptures and that could be followed for at least 56 days (anemia recovery cut-off) (n=83). Pups that died during this period were not included in the study. We recorded the birth date when observed directly or estimated it based on placental and umbilical cord morphology during the first postnatal week (19). At each capture, we recorded sex, body weight, total length, and performed a complete clinical examination. We collected 6 ml of blood from the caudal gluteal or brachial vein using EDTA, heparin, and additive-free vacuum tubes, following previously published protocols for this species (20). We centrifuged additive-free and heparinized blood tubes within 3 hours of blood collection, aliquoted the serum and plasma, and stored them at −20°C in the field (21). We transferred samples to −80°C on the mainland for long-term storage or analysis. We also collected fecal swabs at each capture, placed them in saturated Sheather’s sucrose solution, and processed them in the field laboratory.

### 2.2 Hookworm fecal egg count

We performed fecal egg counts following methods previously validated in this species (20). After removing the swab from the sucrose tube, we added additional Sheather’s solution and placed a glass coverslip on the tube opening. We allowed flotation to occur for one hour, then placed the coverslip onto a glass slide. We scanned the entire cover-slipped area (1200 mm^2^) for hookworm eggs using an optic microscope. In areas with egg aggregation, we counted eggs in 10 randomly selected fields under 100× magnification. We summed the egg counts and reported them as eggs per smear (EPS).

### 2.3 Hematology

We performed complete blood cell counts (CBCs) in the field using EDTA samples and previously validated procedures (21). We manually counted white and red blood cells using a hemocytometer. We also prepared blood smears in the field and stained them later in the mainland laboratory for differential leukocyte counts. We assessed the presence of reticulocytes based on Wright’s Giemsa-stained blood smears by identifying large polychromatic erythrocytes among 1000 erythrocytes examined in the smear monolayer. This was facilitated by the distinct size and polychromasia of reticulocytes in otariids (22). We categorized reticulocyte numbers as adequate if they were > 2% of erythrocytes. We measured hemoglobin (Hb) concentration with the HemoCue™ Hb 201+ system and determined packed cell volume (PCV) by centrifuging hematocrit tubes at 10,000 rpm for 5 minutes. In the mainland lab, we measured serum iron concentration using a ferrozine-based endpoint colorimetric assay.

### 2.4 Cytokine assays

Initially, we assessed thirteen serum cytokines using the CCYTMAG-90K-PX13, MILLIPLEX^®^ Canine Cytokine/Chemokine Magnetic Bead Panel (Premixed 13 Plex - Immunology Multiplex Assay, Millipore Sigma, Merck KGaA, Darmstadt, Germany). The assessed cytokines were granulocyte-macrophage colony-stimulating factor (GM-CSF), interferon-γ (IFN-γ), interleukin-2 (IL-2), interleukin-6 (IL-6), interleukin-7 (IL-7), interleukin-8 (IL-8), interleukin-10 (IL-10), interleukin-15 (IL-15), interleukin-18 (IL-18), interferon-gamma inducible protein 10kDa (IP-10), keratinocyte chemotactic-like (KC-like), monocyte chemoattractant protein-1 (MCP-1), and tumor necrosis factor-α (TNF-α). Canine cytokine reagents were chosen based on known cross-reactivity with northern fur seals (*Callorhinus ursinus*) (23, 24). We ran assays in duplicate using the kit provided reagents. Prior to preparing the reagents, 200 μL of assay buffer was added to each well, then the plate was sealed and shaken at room temperature while preparing the samples, standards, and controls. Since the serum was frozen at -80°C, we warmed the serum samples at room temperature for 15–20 minutes and then centrifuged at 4°C at 10,000x g to remove macro-precipitates. For the validation assays, we initially used 20 μL from the center of each tube, being careful to avoid lipids and the bottom pellet, then diluted with 40 μL of assay buffer (1:2 dilution). We also tested a 1:2 dilution but with a larger volume by using 40 μL of serum and 80 μL of assay buffer. In addition to a larger volume of diluted serum, we added to each well 50 μL of diluted serum rather than 25 μL as instructed in the initial protocol to increase the readability of the samples by the Bio-Plex^®^ 200 system. These modifications of the original protocol improved the R^2^ of the standard curves from an average of 0.91 to 0.97 and the sample coefficient of variation (CV) from an average of 26% to 16% across cytokines. We selected a total of 122 samples from 34 pups for the final cytokine analyses based on the individual pup Hb curves that more reliably allowed to assess anemia recovery.

For preparation of reagents, we followed the protocol provided with the kit, with 10-minute intervals for mixing and dilution being preferred. Once prepared, we incubated the plate overnight at 4°C and shook it at 700x speed. The next morning the remaining protocol was completed. Prior to running the plate, we added wash buffer and shook the plate for five minutes at room temperature. We used the Bio-Plex^®^ 200 system (Bio-Rad Laboratories (Canada) Ltd, Mississauga, Ontario, Canada) to read the plate Bio-Plex Manager™ software to extract the results using the Bio-Plex^®^ 200 system. We used an R^2^ cut-off of 0.95 and a CV cut-off of 15% across assays to select cytokines with more reliable results. Based on these cut-offs we selected seven cytokines (GM-CSF, IFN-γ, IL-6, IL-10, IL-18, MCP-1, and TNF-α) for subsequent analysis.

### 2.5 Data analyses

#### 2.5.1 Classification of anemia and definition of recovery status

We defined anemia as Hb concentrations below 10.1 g/dL, based on biologically relevant thresholds and previously established reference values for this species (mean Hb = 12.26 g/dL; SD = 1.4 g/dL) (21). Pups were categorized as anemic if in any capture point, they had Hb values below the 10.1 g/dL. We further classified anemia into severity categories using standard deviation intervals from the reference mean. We defined mild anemia as 1.6–2.3 SD below the mean (Hb 9.0–10.0 g/dL); moderate anemia as 2.4–3 SD below the mean (Hb 8.0–8.9 g/dL) and severe anemia as more than 3 SD below the mean (Hb < 8.0 g/dL). Anemia was categorized based on mean corpuscular volume (MCV, normocytic or microcytic) and mean corpuscular hemoglobin concentration (MCHC, normochromic or hypochromic) in relation to reference values for pups of this species (MCV mean±SD = 95±16.8; MCHC mean±SD = 31.8±3.5) (21). For pups with ≥3 captures, we assessed recovery from anemia using a cut-off of 56 days of age. We classified pups as “not recovered” if their Hb remained below threshold beyond this age, based on known timelines for hookworm clearance (19, 20).

#### 2.5.2 Hookworm infection and anemia

We defined clinical hookworm infection based on previous studies as the presence of at least one fecal smear with >1 hookworm EPS and any signs of blood in feces (19, 20). We used Fisher’s exact test to compare anemia prevalence between pups with and without clinical hookworm infection. To test the effect of hookworm burden (EPS) on anemia likelihood, we fitted a binomial generalized linear mixed model (GLMM) using the *glmmTMB* package in R sofware (25). We included sex, age and body mass index (BMI, weight/total length) as covariates and used pup ID as a random effect. We evaluated model assumptions using the *DHARMa* package in R software (26).

#### 2.5.3 Hemoglobin curves and predictors of anemia recovery

To test differences in Hb dynamics between anemic and non-anemic pups over the reproductive season, we fitted a GLMM using the pup’s estimated age as a continuous linear predictor associated to a quadratic term and its interaction with anemia categorization as predictors of Hb concentration. We included sex and BMI as fixed effects and treated pup ID as a random effect. We tested different distribution error structures for the response variable based on visual assessment of Hb histograms and comparison of model diagnostic plots, overdispersion and fit (AICc) between models with different response error structures (gamma, log-transformed Gaussian, Gaussian). We selected the Gaussian response of untransformed Hb values based on acceptable diagnostic plots, low overdispersion and lowest AICc.

To evaluate potential associations between BMI, hookworm burden, anemia severity, peripheral blood leukocytes and cytokines with anemia recovery status, we fitted a series of binomial GLMMs using established R packages and diagnostic tools. In all models, the response variable was the pup’s recovery status (yes or no) and pup ID was considered as a random effect. We constructed separate simple models for the following predictors: BMI, hookworm burden, Hb (as a proxy for anemia severity), GM-CSF, IFN-γ, IL-6, IL-10, IL-18, MCP-1, TNF-α, neutrophils, lymphocytes, monocytes, eosinophils, and basophils. We used separate models instead of a single model for all predictors given the high correlation among several predictors (r>0.3, **Supplementary Table 1**) and to avoid overfitting given the constrained sample size (n=122). All these models included age as covariate.

Since GLMMs tested only cross-sectional associations, we further examined whether changes in these predictors prior to recovery (or non-recovery) were more informative. To do this, we calculated the change in each predictor when pups were between 1–2 days-old and 43–55 days-old (before anemia recovery status categorization). We performed this calculation for all pups with sufficient data to assess anemia recovery (n = 24). We then used Wilcoxon rank-sum tests to compare the changes of each predictor between recovery groups. This non-parametric test was chosen due to the small sample size and potential skewness in the data. To control for multiple comparisons, we applied the Benjamini–Hochberg procedure and reported both raw p-values and adjusted q-values to assess statistical significance (27). Next, we fitted a series of binomial generalized linear models (GLMs) to test whether changes in predictors with significant differences between groups (neutrophils, IFN-γ, IL-6, and IL-10) influenced the likelihood of anemia recovery. Due to high correlations among these immune parameters (r > 0.3), we could not include them simultaneously in a single model. Instead, we compared models individually using Akaike’s Information Criterion corrected for small sample size (AICc), implemented via the “MuMIn” R package (28). For this comparison, we only used complete observations across all tested predictors. All models also included the age of the last sampling event for each pup.

To evaluate whether the effects of immune parameters on anemia recovery were related to changes in serum iron, we included serum iron change as an additional predictor in each anemia recovery binomial GLM. We compared model fit (AICc) and examined whether the inclusion of iron altered the effect size or significance of the immune predictors (neutrophils, IFN-γ, IL-6, and IL-10).

Finally, to assess the overall relationship between iron and inflammation, we fitted simple GLMMs with GM-CSF, IFN-γ, IL-6, IL-10, IL-18, MCP-1, TNF-α, neutrophils, lymphocytes, monocytes, eosinophils, and basophils as predictors of serum iron concentrations while controlling for age. We selected a Gaussian distribution for the response based on acceptable diagnostic plots, low overdispersion and lowest AICc compared to models with gamma distribution or log-transformed iron values with a Gaussian error distribution. We included pup ID as a random effect in each model.

## 3 Results

### 3.1 Hookworm infection and characterization of anemia

**Figure 1:**
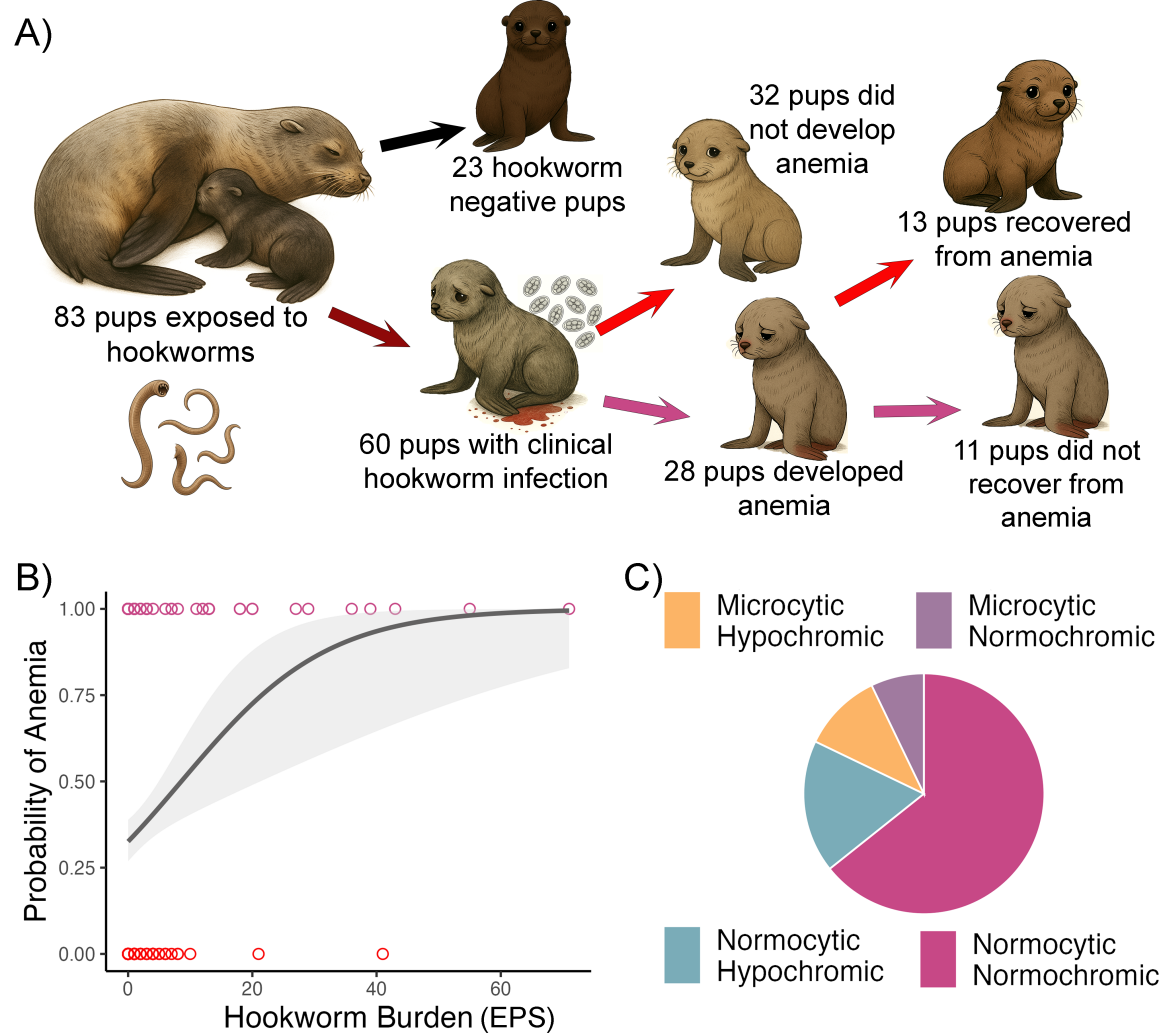
Study design and natural history of hookworm induced anemia in South American fur seal pups. **(A)** Newborn pups are exposed to hookworm larvae through their mother’s colostrum in their first hours of life. Approximately 72% will develop clinical hookworm infection manifesting with hookworm eggs in fecal smears and bloody feces, and ~ 50% of these will develop anemia (Hb <10.1 g/dL). Among 24 pups with repeated assessments, 13 had recovered from anemia by early March whereas 11 pups did not recover from anemia. **(B)** Higher hookworm burden (eggs per fecal smear; EPS) increased the probability of a pup becoming anemic. Plotted raw values (points) and generalized linear model smooth (line) with 95% confidence interval (shade). **(C)** Normocytic normochromic was the most common type of anemia (n=18) followed by normocytic hypochromic (n=5), microcytic hypochromic (n=3), and microcytic normochromic (n=2).

Of the 83 pups included in the study, 60 (72.2%) developed clinical hookworm infection characterized by shedding of hookworm eggs in fecal smears and bloody feces. Among these infected pups, 28 (47.4%) developed anemia, whereas none of the 23 uninfected pups had anemia (Fisher’s exact test, P = 0.0001; **Figure 1A**). Among the 28 anemic pups, we were able to assess anemia recovery status in 24 based on capture dates and estimated age. Thirteen of these 24 pups (54.1%) had recovered from anemia by the end of the study, while 11 pups (45.8%) had not (**Figure 1A**). Anemia severity was mild, moderate or severe in 13 pups, 8 and 7 pups respectively. Hookworm burden had a significant and strong effect on the likelihood of identifying anemia (**Figure 1B**). For each additional egg counted in a fecal smear, pups were 34% more likely to develop anemia (GLMM, OR = 1.34, 95% CI: 1.12–1.62, n = 259). Hookworm burden peaked in mid-January (approximate pup age of 4 weeks) and declined progressively throughout the reproductive season with none of the pups shedding hookworm eggs by the end of the study (**Supplementary Figure 1**). Of the 28 anemic pups, 18 had normocytic normochromic, five had normocytic hypochromic, three had microcytic hypochromic, and two had microcytic normochromic anemia (**Figure 1C**). Interestingly, three pups that initially had normocytic normochromic anemia had microcytic hypochromic anemia at the last capture. All pups had regenerative anemia based on the presence of adequate percentage (>2%) of reticulocytes in the blood smears.

### 3.2 Hemoglobin dynamics and anemia recovery

**Figure 2:**
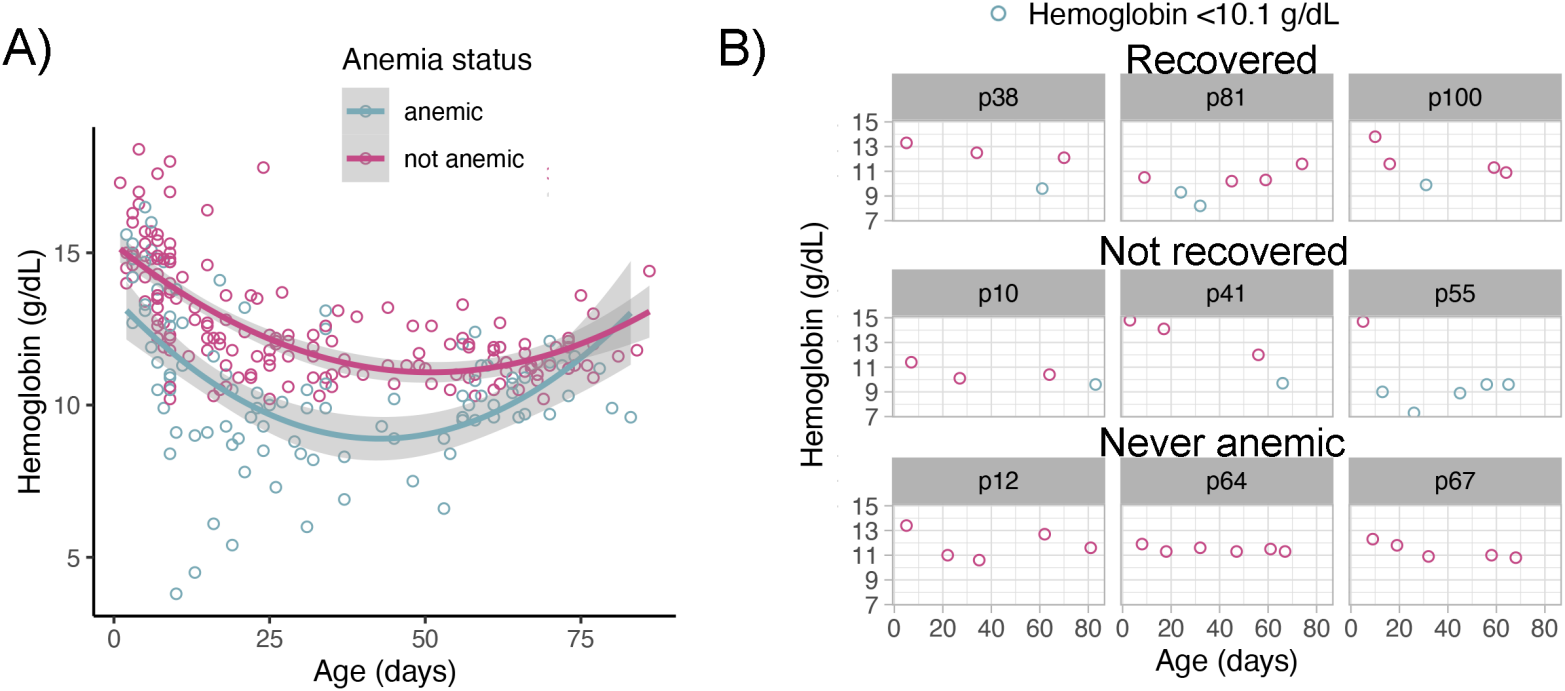
Hemoglobin dynamics in anemic and non-anemic South American fur seal pups. **(A)** Mean hemoglobin concentrations declined in all pups until approximately 50 days of age, thereafter concentrations increased in both groups. Plotted raw hemoglobin values (points) with fitted polynomial generalized linear model smooth (line) with 95% confidence intervals (shade). **(B)** Individual hemoglobin trajectories revealed three distinct patterns: pups that remained non-anemic, pups that developed anemia and recovered, and pups that remained anemic through the end of the study period. Plotted raw hemoglobin values (points). Light blue points represent capture events with hemoglobin values below the anemia threshold (10.1 g/dL). Shaded headers correspond to the pup ID number.

Hemoglobin levels declined in both anemic and non-anemic pups during the first two-thirds of the reproductive season until pups were approximately 50 days old. In the last third of the season, hemoglobin concentrations increased in both groups (GLMM age^2^; β=0.0024±0.00034, Z = 7.13, P<0.001, n = 266) (**Figure 2A**). Although the average hemoglobin curve decline and rise were similar in anemic and non-anemic pups (GLMM, anemia status × age^2^; β=-0.0007±0.0004, Z=-1.7, P = 0.08, n *not-anemic* = 88, n *anemic* = 65), individual hemoglobin trajectories revealed that while some anemic pups recovered by early March, others failed to recover in that timeframe (**Figure 2B**). The rate of hemoglobin decline varied, with some pups showing decreases as steep as –0.150 g/dL/day (median = –0.0647 g/dL/day). Recovery rates ranged from 0.0173 to 0.278 g/dL/day, with some severely anemic pups achieving non-anemic status in < 20 days (mean±SD =29±7 days).

### 3.3 Inflammation impairs recovery from anemia

**Figure 3:**
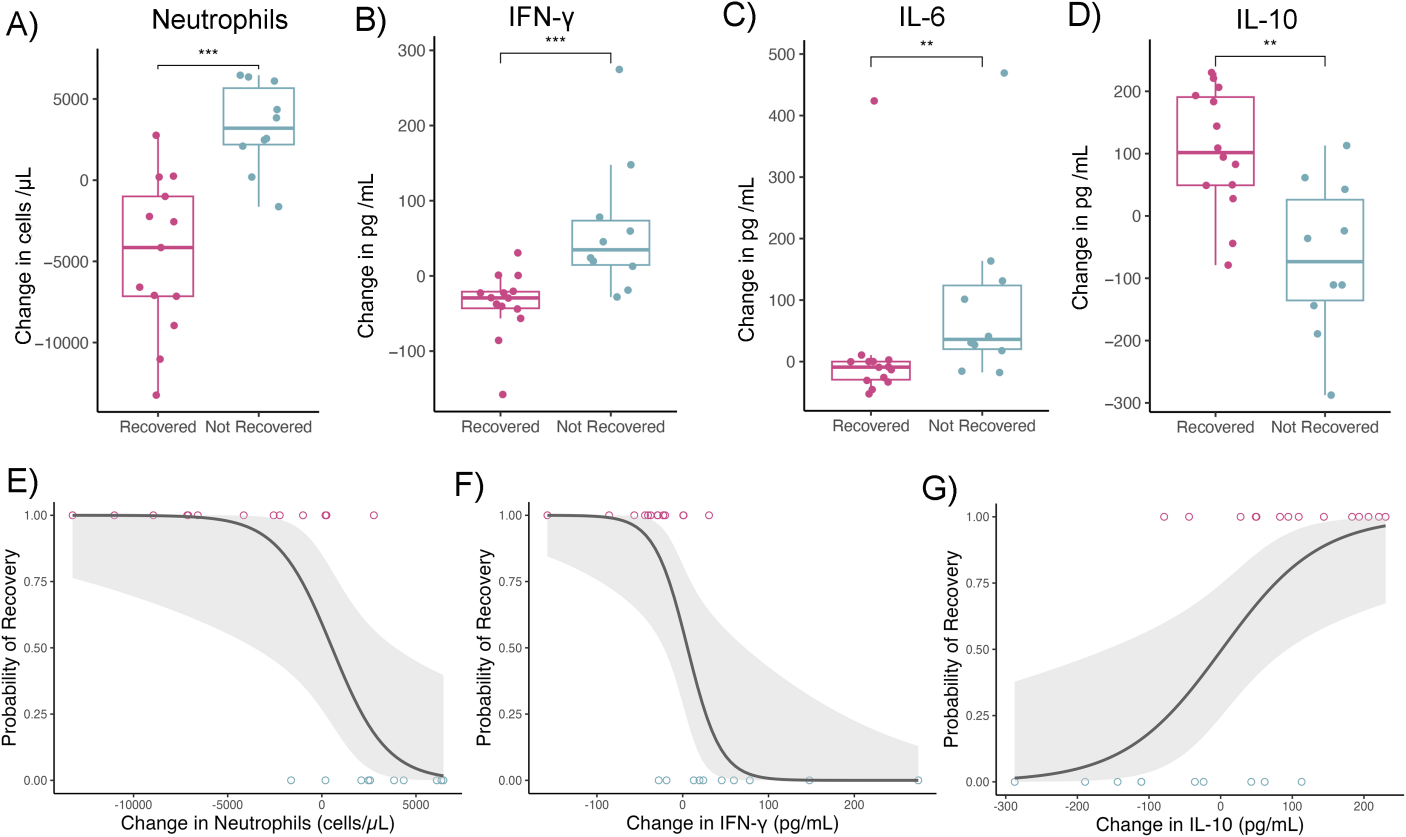
Systemic inflammation reduces the likelihood of anemia recovery in fur seal pups. **(A–D)** Boxplots show the change in circulating cytokines and cells between the first and subsequent capture. On average, pups that recovered from anemia showed a decline in **(A)** neutrophil count, **(B)** IFN-γ, and **(C)** IL-6 concentration, while pups that did not recover had increases in these markers. In contrast, **(D)** IL-10 concentration increased in most recovered pups but declined in non-recovered pups. Boxplot with box containing the median, 25%, 75% quartiles and whisker representing 95% percentiles (n = 24). Points represent raw cytokine values. Asterisks indicate statistically significant differences between groups (Mann-Whitney test with posthoc p-value correction for multiple hypothesis testing. ***P<0.001; **P=0.001 - 0.01). **(E–G)** Scatterplots illustrate the association between changes in inflammatory mediators and effector cells on the probability of anemia recovery. Increases in **(E)** neutrophils and **(F)** IFN-γ were associated with a lower likelihood of recovery, while **(G)** increases in IL-10 were associated with a higher probability of recovery. Plotted raw values (points) and generalized linear model smooth (line) with 95% confidence interval (shade) (n = 24).

Average pup BMI, anemia severity, hookworm burden, cytokines and peripheral blood leukocytes were not associated with anemia recovery status (**Supplementary Table 2**). Therefore, we examined whether changes in hookworm burden, body mass (growth rate), and immune analytes prior to classification as “recovered” or “not recovered” could predict outcomes, under the hypothesis that pathophysiological shifts precede recovery trajectories rather than simply co-vary with them. Changes in anemia severity, hookworm burden, and body mass did not differ between recovered and non-recovered pups (**Supplementary Table 3**). However, pups that did not recover from anemia showed increases in neutrophil count and plasma concentration of IFN-γ and IL-6 relative to their baseline values, whereas pups that recovered had declines in these markers (**Figure 3**). In contrast, IL-10 concentration increased in most recovered pups and declined in non-recovered pups (**Figure 3**). These patterns suggest that sustained activation of systemic inflammation impairs anemia resolution. For instance, each 1 pg/mL increase in IFN-γ was associated with a 5.3% higher likelihood of not recovering from anemia (GLM; OR 95% CI = 1.02–1.11, n = 24), and each increase in 100 neutrophils/μL in blood corresponded to an 8.0% higher likelihood of non-recovery (GLM; OR 95% CI = 1.0003–1.0019, n = 24).

Due to co-linearity among inflammatory markers, additive models were not feasible; however, model selection analyses identified rises in neutrophils and IFN-γ and declines in IL-10 as significant predictors of non-recovery (**Table 1**). These effects were independent of changes in serum iron concentrations, as the inclusion of iron did not alter model significance or affect odds ratios (**Supplementary Table 3**). IL-6 was the only inflammatory marker marginally associated with iron status, with increased IL-6 corresponding to reduced serum iron (GLMM, β = –0.011 ± 0.0058, Z = –1.98, P = 0.0469, n = 120).

**Table 1:** Ranking of binomial generalized linear models for anemia recovery. Coefficients for the effects of changes in IFN-γ, IL-6, IL-10 and neutrophils on the likelihood of recovery from anemia.

Model	(Intercept)	IFN-γ	IL-6	IL-10	Neutrophils	df	logLik	AICc	delta	weight
1	10.10759				0.000799	3	-5.32	17.91	0	0.9567
2	0.724244	0.052073				3	-8.78	24.76	6.9	0.0311
3	-4.51204			-0.01839		3	-9.72	26.64	8.7	0.0121
4	1.281051		0.005165			3	-14.96	37.12	19.2	0.0001

Models were ranked based on corrected Akaike’ Information Criteria. * = P<0.05. n = 24. Models also control for the effect of age.

## 4 Discussion

In the context of hematophagous parasite infection, anemia has traditionally been considered a direct consequence of parasite-induced blood loss (16, 29, 30). However, conversely to conclusions from experimental models (31, 32), inflammation could sustain anemia in naturally evolved hematophagous parasite-host relationships as a strategy to reallocate host resources (6, 10). In support, our findings reveal that the host inflammatory response can significantly influence the resolution of anemia. Specifically, South American fur seal pups that failed to recover from hookworm-associated anemia had sustained higher concentration of neutrophils, IFN-γ, and IL-6, while those that recovered showed decreases in these markers and increased IL-10 concentration. These patterns suggest that systemic inflammation may impair recovery from anemia, potentially directing host resources away from erythropoiesis.

Hookworms are a common cause of anemia in both humans and animals (16, 29, 30). Some individuals fail to recover from anemia even after the infection is cleared, with or without pharmacological intervention (33). The reasons for this incomplete recovery remain unclear but may involve lack of iron stores, insufficient caloric intake, or impaired absorption of essential nutrients due to gut damage, effects usually seen only with high hookworm burdens (29, 34). In our study, although hookworm burden predicted the development of anemia, it did not predict recovery. Likewise, body mass, growth rate, and serum iron concentration were not associated with recovery, suggesting these factors have a limited role in the resolution of anemia post hookworm infection in fur seals.

Compared to terrestrial mammals, fur seals are particularly efficient at eliminating hookworms (19), and therefore, our study focused on these pups that survive hookworm disease and successfully clear the infection. Although some individuals were categorized as either recovered or not recovered from anemia, their individual hemoglobin trajectories were either flat or increasing by the end of the study. This suggests that with a longer observation period, most (if not all) pups would have likely recovered from anemia. However, our findings indicate that approximately half of the anemic pups recover more rapidly, while the other half had delayed recovery, likely due to sustained systemic inflammation. This delay may be immediately detrimental given the high immunopathological cost of inflammation in early life (35), evidenced as prolonged anemia in our study. Lower hemoglobin concentration has been associated with postponed development of swimming and diving skills (13), abilities that are essential for foraging and weaning success in otariids (36). Therefore, systemic inflammation may impose fitness costs in the short term. However, if delayed anemia recovery due to systemic inflammation was purely disadvantageous this phenotype would not have been preserved through natural selection. Ecological trade-off theory offers a possible explanation: under certain conditions, costs incurred in one domain may be offset by future benefits, maintaining an evolutionarily stable strategy (6, 37, 38). In the case of fur seal pups, while systemic inflammation may prolong anemia and delay diving competency, it could enhance or decrease immune defense against other pathogens. The polarization of immune system to confront a helminth infection (a Th2 response) can affect the immune response to bacterial or viral agents (Th1 and Th17 responses) (39). This immune polarization can either increase or decrease the host’s susceptibility to bacterial and viral infections (40, 41). Congruent with this statement, following hookworm clearance, pups are at risk of developing a respiratory syndrome linked to mites and various bacterial species (42). One possible explanation is that these secondary infections arise because the immune response to hookworm increases the susceptibility of fur seal pups. Our preliminary findings over three reproductive seasons suggest that pups with prolonged inflammation are less likely to develop this syndrome (43). Therefore, it is likely that systemic inflammation might redirect physiological resources from hematopoiesis toward enhanced immune defense against other microorganisms. This pattern could reflect a tolerance–resistance trade-off for respiratory infection, a concept well established in ecoimmunology (44, 45). Theory and empirical evidence suggest that the relative costs and benefits of these strategies depend on life-history traits (e.g., reproductive rate) and ecological conditions (e.g., resource availability) (45, 46). Future work should test whether such forces shape the balance between tolerance and resistance in fur seal pups. Why some pups maintain a proinflammatory state and recover slower from anemia remains an open question, but host, pathogen and environmental factors likely play a role. For the host, the impact of genotypes could be a significant factor since it has been demonstrated in our studied species and other otariids that overall genetic diversity enhances resistance to hookworm disease (47, 48). Additionally, the inflammatory response exhibits significant variability between individuals. In humans, inter-individual inflammatory variability is associated with the genetic and microbial background (49, 50). Therefore, the genetic makeup and microbiota of seal pups are likely to determine the magnitude of the inflammatory response and, consequently, the pups’ ability to recover from the infection. On the pathogen side, sustained inflammation could be triggered by exposure to immunogenic microbes. The intestinal damage caused by hookworms facilitates systemic bacterial translocation from the gut (51, 52), which could be one potential trigger for sustained inflammation in some pups, delaying anemia recovery. Finally, milk nutritional or immunological factors, known regulators of inflammation in mammalian neonates (53, 54), could have also played a role on determining inflammatory profiles in pups that recover faster and pups that did not recover from anemia.

Our inflammation and anemia findings align with a growing body of literature demonstrating the immunopathological costs of inflammation in early life. In neonatal mammals, inflammatory signals such as IFN-γ and IL-6 can inhibit differentiation of erythroid progenitor cells, disrupt iron recycling, and sequester iron within macrophages, leading to functional iron deficiency (3). This mechanism is well described in clinical and laboratory settings, but its relevance in wild animal populations has remained speculative (55, 56). Interestingly, in our study, the effects of inflammation on anemia recovery were independent of iron serum concentration. However, iron plasma concentration by itself is only a partial reflection of iron metabolism, and mammals can have functional iron deficiency with depleted iron storages but normal iron plasma concentration (57). In our study system, most pups had normocytic normochromic anemia, which can be associated with functional iron deficiency (58). Furthermore, at least three pups transitioned to microcytic hypochromic anemia, the stereotypical anemia type associated with advanced iron deficiency (58). Fur seal pups are rapidly growing during the first 2 months of life, which implies high iron demands. In this context, the blood loss due to parasitism and potential disruption of iron metabolism by inflammation could be the “perfect storm” that places pups at risk of functional iron deficiency. Alternatively, we cannot rule out that young diving mammals have alternative mechanisms of iron regulation that protects them from functional deficiency during inflammatory anemia.

Besides disruption of iron metabolism, inflammation can perpetuate anemia through disruption of erythroid precursor differentiation and stimulation of erythrophagocytosis. In murine models, IFN-γ directly inhibited erythroid colony formation and promoted macrophage activation, which increased phagocytosis of red blood cells (59, 60). If similar processes occur in fur seals, it could explain the negative effect of early increases in IFN-γ on anemia recovery. Similarly, the rise in neutrophils in fur seal pups likely indicated increased granulopoiesis due to inflammation heralded by increased IFN-γ and other inflammatory cytokines (3). This could explain the strength of an increase in neutrophils as a negative predictor of anemia recovery in fur seal pups.

Interestingly, IL-10 emerged as a potential protective factor in pups that recovered from anemia. This cytokine exerts a dual role in regulating inflammatory responses and erythropoiesis. In humans, for instance, IL-10 administration in therapeutic settings impaired erythropoiesis and exacerbated inflammatory anemia (61, 62). However, in field studies, declining or dysregulated IL-10 have been implicated in the development of severe anemia during infectious diseases such as malaria (63). This apparent dichotomy may be explained by differential IL-10 receptor (IL-10R) engagement. When IL-10 binds predominantly to low-affinity IL-10R β-chains, as is likely the case during pharmacological administration, it may paradoxically enhance inflammatory signaling. In contrast, IL-10 signaling through high-affinity IL-10R α-chain promotes classic anti-inflammatory responses (64). In our study, IL-10 differed from pro-inflammatory cytokines such as IL-6 and IFN-γ, consistent with a regulatory effect. Furthermore, the positive association of IL-10 with anemia recovery supports the notion that immune regulation contributes to the resolution of anemia in fur seal pups, mirroring findings from infections of humans with hematophagous parasites.

The study of immunity in wild animals provides valuable insight into fundamental and evolutionarily conserved mechanisms of disease regulation (65). However, such studies also face unique challenges that can introduce limitations, as was the case here. Our sample size was limited by the challenges of long-term monitoring and recapture, which reduced power to detect subtle interactions. We also relied on peripheral blood cytokines as proxies of systemic responses, which may miss tissue-specific dynamics. Future work should extend longitudinal follow-up beyond 12 weeks and incorporate transcriptomic or proteomic profiling—particularly in hematopoietic and mucosal tissues that directly interact with pathogens—to clarify mechanism and fitness consequences. In addition, genomic characterization of pups (e.g., genome-wide genotyping or targeted sequencing of cytokine/PRR and iron-regulatory pathways, including hepcidin) could help to evaluate whether the observed inflammatory phenotypes reflect adaptive differences. Because plasma iron alone may not diagnose iron deficiency, iron status should be assessed with a panel including transferrin saturation and ferritin. Finally, to evaluate persistent danger signaling after worm clearance, low-biomass blood microbial profiling with rigorous contamination controls and complementary markers of microbial translocation (e.g., LBP, sCD14) could be attempted in similar studies. Together, these approaches will help discriminate among mechanisms driving prolonged inflammation and delayed anemia recovery.

This study aimed to determine whether systemic inflammation induced by parasitic infection impairs recovery from anemia in a naturally co-evolved host–parasite system. Through integrated assessments of health status, immune function, and iron levels in wild fur seal pups, we found that sustained inflammation, rather than parasite burden or iron availability, was the primary factor associated with anemia recovery. These findings highlight a physiological cost of immune activation: delayed resolution of anemia during a critical developmental window, a trade-off likely shaped by evolutionary pressures in pathogen-rich environments. Wild animals, like humans, have evolved under complex ecological pressures, where exposure to multiple infections exerts strong selective forces (66, 67). In this context, trade-offs between anemia recovery and immune activation may reflect evolutionarily conserved strategies shaped by natural selection. Such trade-offs could help explain the preservation of fundamental physiological links between inflammation and anemia across diverse mammalian taxa, including diving mammals, for whom the costs of anemia may be especially high.

## Data Availability

The original contributions presented in the study are included in the article/**Supplementary Material**. Further inquiries can be directed to the corresponding author/s.

## References

[1] Osterholm EA Georgieff MK . Chronic inflammation and iron metabolism. J Pediatr. (2015) 166:1351–1357.e1. doi: 10.1016/j.jpeds.2015.01.017, PMID: 25684086 PMC4446233

[2] German KR Juul SE . Neonatal anemia. Curr Pediatr Rev. (2023) 19:388–94. doi: 10.2174/1573396319666221121140627, PMID: 36411551

[3] Canny SP Orozco SL Thulin NK Hamerman JA . Immune mechanisms in inflammatory anemia. Annu Rev Immunol. (2023) 41:405–29. doi: 10.1146/annurev-immunol-101320-125839, PMID: 36750316 PMC10367595

[4] Li M Li W Liu Y Yin X Fan MZ . 437 Effects of lipopolysaccharide challenge and weaning on serum biochemical parameters and hepatic hepcidin gene expression in piglets. J Anim Sci. (2017) 95:214–4. doi: 10.2527/asasann.2017.437

[5] Sickinger M Joerling J Büttner K Roth J Wehrend A . Influence of an iron dextran injection in various diseases on hematological blood parameters, including serum ferritin, neonatal dairy calves. BMC Vet Res. (2024) 20:379. doi: 10.1186/s12917-024-04229-y, PMID: 39182079 PMC11344462

[6] Budischak SA Wiria AE Hamid F Wammes LJ Kaisar MMM Van Lieshout L . Competing for blood: the ecology of parasite resource competition in human malaria–helminth co-infections. Ecol Lett. (2018) 21:536–45. doi: 10.1111/ele.12919, PMID: 29417715

[7] Pedersen AB Fenton A . Wild rodents as a natural model to study within-host parasite interactions. Wildl Dis Ecol Link Theory Data Appl. (2019), 58–90. doi: 10.1017/9781316479964.003

[8] Hotez PJ Ghosh K Huwdon J Narasimhan S Jones B Shuhua X . Experimental approaches to the development of a recombinant hookworm vaccine. Immunol Rev. (1999) 171:163–71. doi: 10.1111/j.1600-065X.1999.tb01347.x, PMID: 10582170

[9] Mendez S Zhan B Goud G Ghosh K Dobardzic A Wu W . Effect of combining the larval antigens Ancylostoma secreted protein 2 (ASP-2) and metalloprotease 1 (MTP-1) in protecting hamsters against hookworm infection and disease caused by *Ancylostoma ceylanicum*. Vaccine. (2005) 23:3123–30. doi: 10.1016/j.vaccine.2004.12.022, PMID: 15837211

[10] Budischak SA Jolles AE Ezenwa VO . Direct and indirect costs of co-infection in the wild: Linking gastrointestinal parasite communities, host hematology, and immune function. Int J Parasitol Parasites Wildl. (2012) 1:2–12. doi: 10.1016/j.ijppaw.2012.10.001, PMID: 24533308 PMC3904086

[11] Rosen DAS Hindle AG Gerlinsky CD Goundie E Hastie GD Volpov BL . Physiological constraints and energetic costs of diving behaviour in marine mammals: a review of studies using trained Steller sea lions diving in the open ocean. J Comp Physiol B. (2017) 187:29–50. doi: 10.1007/s00360-016-1035-8, PMID: 27686668

[12] Kanatous SB DiMichele LV Cowan DF Davis RW . High aerobic capacities in the skeletal muscles of pinnipeds: adaptations to diving hypoxia. J Appl Physiol. (1999) 86:1247–56. doi: 10.1152/jappl.1999.86.4.1247, PMID: 10194210

[13] Montalva F Pérez-Venegas D Gutiérrez J Seguel M . The contrasting hidden consequences of parasitism: Effects of a hematophagous nematode (*Uncinaria* sp.) in the development of a marine mammal swimming behavior. Ecol Evol. (2019) 9:1–11. doi: 10.1002/ece3.4914, PMID: 31015959 PMC6468065

[14] Villegas-Amtmann S Atkinson S Paras-Garcia A Costa DP . Seasonal variation in blood and muscle oxygen stores attributed to diving behavior, environmental temperature and pregnancy in a marine predator, the California sea lion. Comp Biochem Physiol A Mol Integr Physiol. (2012) 162:413–20. doi: 10.1016/j.cbpa.2012.04.019, PMID: 22561663

[15] Lindsay SA Fulham M Caraguel CGB Gray R . Mitigating disease risk in an endangered pinniped: early hookworm elimination optimizes the growth and health of Australian sea lion pups. Front Vet Sci. (2023) 10:1161185. doi: 10.3389/fvets.2023.1161185, PMID: 37180065 PMC10168540

[16] Seguel M Gottdenker N . The diversity and impact of hookworm infections in wildlife. Int J Parasitol Parasites Wildl. (2017) 6:177–94. doi: 10.1016/j.ijppaw.2017.03.007, PMID: 28765810 PMC5526439

[17] Lyons ET Spraker TR De Long RL Ionita M Melin SR Nadler SA . Review of research on hookworms (*Uncinaria lucasi* Stiles, 1901) in northern fur seals (*Callorhinus ursinus* Linnaeus, 1758). Parasitol Res. (2011) 109:257–65. doi: 10.1007/s00436-011-2420-6, PMID: 21537983

[18] Marcus AD Higgins DP Gray R . Epidemiology of hookworm (*Uncinaria sanguinis*) infection in free-ranging Australian sea lion (*Neophoca cinerea*) pups. Parasitol Res. (2014) 113:3341–53. doi: 10.1007/s00436-014-3997-3, PMID: 25056940

[19] Seguel M Montalva F Perez-Venegas D Gutiérrez J Paves HJ Müller A . Immune-mediated hookworm clearance and survival of a marine mammal decrease with warmer ocean temperatures. eLife. (2018) 7:e38432. doi: 10.7554/eLife.38432, PMID: 30398149 PMC6245726

[20] Seguel M Muñoz F Perez-Venegas D Müller A Paves H Howerth E . The life history strategy of a fur seal hookworm in relation to pathogenicity and host health status. Int J Parasitol Parasites Wildl. (2018) 7:251–60. doi: 10.1016/j.ijppaw.2018.07.003, PMID: 30069428 PMC6067062

[21] Seguel M Muñoz F Keenan A Perez-Venegas D DeRango G Pavés H . Hematology, serum chemistry, and early hematologic changes in free-ranging South American fur seals (*Arctocephalus australis*) at Guafo Island, Chilean Patagonia. J Wildl Dis. (2016) 52:663–8. doi: 10.7589/2015-11-293, PMID: 27243331

[22] Flores-Morán A Banuet-Martínez M Elorriaga-Verplancken FR García-Ortuño LE Sandoval-Sierra J Acevedo-Whitehouse K . Atypical red blood cells are prevalent in California sea lion pups born during anomalous sea surface temperature events. Physiol Biochem Zool. (2017) 90:564–74. doi: 10.1086/692919, PMID: 28671858

[23] Cerda M-IM Gray R Higgins DP . Cytokine RT-qPCR and ddPCR for immunological investigations of the endangered Australian sea lion (*Neophoca cinerea*) and other mammals. PeerJ. (2020) 8:e10306. doi: 10.7717/peerj.10306, PMID: 33240637 PMC7668205

[24] Johnson V Moore AR Conway R Zeppelin T Gelatt T Duncan C . Establishing a reference interval for acute phase proteins, cytokines, antioxidants and commonly measured biochemical and hematologic parameters in the northern fur seal (*Callorhinus ursinus*). Vet Immunol Immunopathol. (2021) 242:110348. doi: 10.1016/j.vetimm.2021.110348, PMID: 34689000

[25] Brooks ME Kristensen K Van Benthem KJ Magnusson A Berg CW Nielsen A . glmmTMB balances speed and flexibility among packages for zero-inflated generalized linear mixed modeling. R J. (2017) 9:378–400. doi: 10.32614/rj-2017-066

[26] Hartig F . DHARMa: residual diagnostics for hierarchical (multi-level/mixed) regression models (2019). R package version 0.4.7. Available online at: https://CRAN.R-project.org/package=DHARMa. (Accessed January 13, 2025)

[27] Verhoeven KJF Simonsen KL McIntyre LM . Implementing false discovery rate control: increasing your power. Oikos. (2005) 108:643–7. doi: 10.1111/j.0030-1299.2005.13727.x

[28] Barton K . Multi-Model Inference. (2018), R package version 1.42.1. Available online at: https://CRAN.R-project.org/package=MuMIn. (Accessed January 20, 2025)

[29] Loukas A Hotez PJ Diemert D Yazdanbakhsh M McCarthy JS Correa-Oliveira R . Hookworm infection. Nat Rev Dis Primer. (2016) 2:1–18. doi: 10.1038/nrdp.2016.88, PMID: 27929101

[30] Pawlowski ZS SChad GA Stott GJ Organization WH . Hookworm infection and anaemia : approaches to prevention and control (1991). World Health Organization. Available online at: https://apps.who.int/iris/handle/10665/40857 (Accessed October 11, 2021).

[31] Ferguson AA Inclan-Rico JM Lu D Bobardt SD Hung L Gouil Q . Hookworms dynamically respond to loss of Type 2 immune pressure. PloS Pathog. (2023) 19:e1011797. doi: 10.1371/journal.ppat.1011797, PMID: 38079450 PMC10735188

[32] Furtado LFV Alves WP Da Silva VJ Rabelo ÉML . Hookworm infection as a model for deepen knowledge of iron metabolism and erythropoiesis in anemia. Cytokine. (2024) 177:156559. doi: 10.1016/j.cyto.2024.156559, PMID: 38412767

[33] Rohner F Zimmermann MB Amon RJ Vounatsou P Tschannen AB N’Goran EK . In a randomized controlled trial of iron fortification, anthelmintic treatment, and intermittent preventive treatment of malaria for anemia control in ivorian children, only anthelmintic treatment shows modest benefit. J Nutr. (2010) 140:635–41. doi: 10.3945/jn.109.114256, PMID: 20107144

[34] Bartsch SM Hotez PJ Asti L Zapf KM Bottazzi ME Diemert DJ . The global economic and health burden of human hookworm infection. PloS Negl Trop Dis. (2016) 10:e0004922. doi: 10.1371/journal.pntd.0004922, PMID: 27607360 PMC5015833

[35] Khan I Agashe D Rolff J . Early-life inflammation, immune response and ageing. Proc R Soc B Biol Sci. (2017) 284:20170125. doi: 10.1098/rspb.2017.0125, PMID: 28275145 PMC5360934

[36] Schwarz JFL DeRango EJ Zenth F Kalberer S Hoffman JI Mews S . A stable foraging polymorphism buffers Galápagos sea lions against environmental change. Curr Biol. (2022) 32:1623–1628.e3. doi: 10.1016/j.cub.2022.02.007, PMID: 35240048

[37] Farahpour F Saeedghalati M Brauer VS Hoffmann D . Trade-off shapes diversity in eco-evolutionary dynamics. eLife. (2018) 7:e36273. doi: 10.7554/eLife.36273, PMID: 30117415 PMC6126925

[38] Hawley DM Altizer SM . Disease ecology meets ecological immunology: Understanding the links between organismal immunity and infection dynamics in natural populations. Funct Ecol. (2011) 25:48–60. doi: 10.1111/j.1365-2435.2010.01753.x

[39] Chen H Cao Z Liu M Diamond MS Jin X . The impact of helminth-induced immunity on infection with bacteria or viruses. Vet Res. (2023) 54:87. doi: 10.1186/s13567-023-01216-3, PMID: 37789420 PMC10548622

[40] Desai P Diamond MS Thackray LB . Helminth–virus interactions: determinants of coinfection outcomes. Gut Microbes. (2021) 13:1961202. doi: 10.1080/19490976.2021.1961202, PMID: 34428107 PMC8405156

[41] Lin JS Mohrs K Szaba FM Kummer LW Leadbetter EA Mohrs M . Virtual memory CD8 T cells expanded by helminth infection confer broad protection against bacterial infection. Mucosal Immunol. (2019) 12:258–64. doi: 10.1038/s41385-018-0100-x, PMID: 30361537 PMC6301144

[42] Seguel M Gutiérrez J Hernández C Montalva F Verdugo C . Respiratory mites (*Orthohalarachne diminuata*) and β-hemolytic Streptococci-associated bronchopneumonia outbreak in south American fur seal pups (*Arctocephalus australis*). J Wildl Dis. (2018) 54:380–5. doi: 10.7589/2017-09-214, PMID: 29369727

[43] Co C . Investigation of neonatal hookworm (*Uncinaria* sp.) infection on immune variation in south american fur seal pups (*Arctocephalis australis*) (2024). Guelph, Ontario: University of Guelph. Available online at: https://atrium.lib.uoguelph.ca/items/afb61b31-1ea5-4f6d-9415-974320ac1aaa. (Accessed January 13, 2025)

[44] Råberg L . How to live with the enemy: understanding tolerance to parasites. PloS Biol. (2014) 12:e1001989. doi: 10.1371/journal.pbio.1001989, PMID: 25369060 PMC4219658

[45] Ahmad HI Jabbar A Mushtaq N Javed Z Hayyat MU Bashir J . Immune tolerance vs. immune resistance: the interaction between host and pathogens in infectious diseases. Front Vet Sci. (2022) 9:827407. doi: 10.3389/fvets.2022.827407, PMID: 35425833 PMC9001959

[46] Howick VM Lazzaro BP . Genotype and diet shape resistance and tolerance across distinct phases of bacterial infection. BMC Evol Biol. (2014) 14:56. doi: 10.1186/1471-2148-14-56, PMID: 24655914 PMC3997931

[47] Gutiérrez J Seguel M Saenz-Agudelo P Acosta-Jamett G Verdugo C . Host genetic diversity and body condition influence parasite resistance and clearance in a wild marine mammal population. Biol Lett. (2024) 20:20240302. doi: 10.1098/rsbl.2024.0302, PMID: 39353568 PMC11444764

[48] Acevedo-Whitehouse K Spraker TR Lyons E Melin SR Gulland F Delong RL . Contrasting effects of heterozygosity on survival and hookworm resistance in California sea lion pups. Mol Ecol. (2006) 15:1973–82. doi: 10.1111/j.1365-294X.2006.02903.x, PMID: 16689912

[49] Li Y Oosting M Smeekens SP Jaeger M Aguirre-Gamboa R Le KTT . A functional genomics approach to understand variation in cytokine production in humans. Cell. (2016) 167:1099–1110.e14. doi: 10.1016/j.cell.2016.10.017, PMID: 27814507

[50] Schirmer M Smeekens SP Vlamakis H Jaeger M Oosting M Franzosa EA . Linking the human gut microbiome to inflammatory cytokine production capacity. Cell. (2016) 167:1125–1136.e8. doi: 10.1016/j.cell.2016.10.020, PMID: 27814509 PMC5131922

[51] George PJ Anuradha R Kumar NP Kumaraswami V Nutman TB Babu S . Evidence of microbial translocation associated with perturbations in t cell and antigen-presenting cell homeostasis in hookworm infections. PloS Negl Trop Dis. (2012) 6:e1830. doi: 10.1371/journal.pntd.0001830, PMID: 23056659 PMC3464301

[52] Seguel M Muñoz F Navarrete MJ Paredes E Howerth E Gottdenker N . Hookworm Infection in south american fur seal (*Arctocephalus australis*) pups. Vet Pathol. (2017) 54:288–97. doi: 10.1177/0300985816677151, PMID: 28207376

[53] Andreas NJ Kampmann B Mehring Le-Doare K . Human breast milk: A review on its composition and bioactivity. Early Hum Dev. (2015) 91:629–35. doi: 10.1016/j.earlhumdev.2015.08.013, PMID: 26375355

[54] Peetsalu K Niine T Loch M Dorbek-Kolin E Tummeleht L Orro T . Effect of colostrum on the acute-phase response in neonatal dairy calves. J Dairy Sci. (2022) 105:6207–19. doi: 10.3168/jds.2021-21562, PMID: 35534273

[55] Harvey JW Harr KE Murphy D Walsh MT De Wit M Deutsch CJ . Serum Iron analytes in healthy and diseased florida manatees (*Trichechus manatus latirostris*). J Comp Pathol. (2019) 173:58–70. doi: 10.1016/j.jcpa.2019.10.006, PMID: 31812174

[56] Steyrer C Miller M Hewlett J Buss P Hooijberg EH . Markers of inflammation in free-living African elephants (*Loxodonta africana*): Reference intervals and diagnostic performance of acute phase reactants. Vet Clin Pathol. (2023) 52:75–86. doi: 10.1111/vcp.13197, PMID: 36303463

[57] Ganz T . Anemia of inflammation. N Engl J Med. (2019) 381:1148–57. doi: 10.1056/NEJMra1804281, PMID: 31532961

[58] Radakovich LB Olver CS . Iron and copper deficiencies, and disorders of iron metabolism. In: Schalm’s Veterinary Hematology. Hoboken, NJ, USA: John Wiley & Sons, Ltd (2022). p. 215–20. doi: 10.1002/9781119500537.ch27

[59] Merli P Quintarelli C Strocchio L Locatelli F . The role of interferon-gamma and its signaling pathway in pediatric hematological disorders. Pediatr Blood Cancer. (2021) 68:e28900. doi: 10.1002/pbc.28900, PMID: 33484058

[60] Cnops J De Trez C Stijlemans B Keirsse J Kauffmann F Barkhuizen M . NK-, NKT- and CD8-derived IFNγ drives myeloid cell activation and erythrophagocytosis, resulting in trypanosomosis-associated acute anemia. PLoS Pathog. (2015) 11:e1004964. doi: 10.1371/journal.ppat.1004964, PMID: 26070118 PMC4466398

[61] Tilg H Ulmer H Kaser A Weiss G . Role of IL-10 for induction of anemia during inflammation. J Immunol. (2002) 169:2204–9. doi: 10.4049/jimmunol.169.4.2204, PMID: 12165551

[62] Saraiva M Vieira P O’Garra A . Biology and therapeutic potential of interleukin-10. J Exp Med. (2020) 217:e20190418. doi: 10.1084/jem.20190418, PMID: 31611251 PMC7037253

[63] Boeuf PS Loizon S Awandare GA Tetteh JK Addae MM Adjei GO . Insights into deregulated TNF and IL-10 production in malaria: implications for understanding severe malarial anaemia. Malar J. (2012) 11:253. doi: 10.1186/1475-2875-11-253, PMID: 22853732 PMC3469355

[64] Saxton RA Tsutsumi N Su LL Abhiraman GC Mohan K Henneberg LT . Structure-based decoupling of the pro- and anti-inflammatory functions of interleukin-10. Science. (2021) 371:eabc8433. doi: 10.1126/science.abc8433, PMID: 33737461 PMC9132103

[65] Flies AS . Rewilding immunology Integrating comparative immunology can improve human, animal, and ecosystem health. Science. (2020) 369:37–8. doi: 10.1126/science.abb8664, PMID: 32631885

[66] Karlsson EK Kwiatkowski DP Sabeti PC . Natural selection and infectious disease in human populations. Nat Rev Genet. (2014) 15:379–93. doi: 10.1038/nrg3734, PMID: 24776769 PMC4912034

[67] Lane-Degraaf KE Amish SJ Gardipee F Jolles A Luikart G Ezenwa VO . Signatures of natural and unnatural selection: evidence from an immune system gene in African buffalo. Conserv Genet. (2015) 16:289–300. doi: 10.1007/s10592-014-0658-0

