# Supplementary material for: Systemic inflammation impairs recovery from hookworm-associated anemia in a wild marine mammal host
Source: Front Immunol. 2025 Nov 11;16:1659969. doi: 10.3389/fimmu.2025.1659969 (PMC12643882; doi:10.3389/fimmu.2025.1659969)
Supplement: Supplementary file 2 [file DataSheet2.docx]

Supplementary Materials

Systemic inflammation impairs recovery from hookworm-associated anemia in a wild marine mammal host

Violetta Zaitseva^1^, Nanami Arakawa^1^, Carmon Co^1^, Aranza Gomez-Camus^1,9^, Diego Perez-Venegas^2,3^, Felipe Montalva^3^, Josefina Gutierrez^3,4,5^, Claudia Ulloa-Contreras^6^, Ricardo Chihuailaf ^7^, Claudio Verdugo^4,8^, Dorothee Bienzle^1^, Mauricio Seguel*^1,3,9^

Supplementary table 1. Correlation matrix of immunological and physiological parameters in South American fur seal pups. Upper corner contain Spearman rho coefficients and lower corner p-values.

|  | body mass index | hookworm eggs | Hemoglobin | neutrophils | lymphocytes | monocytes | eosinophils | basophils | GM-CSF | IFNγ | IL-6 | IL-10 | IL-18 | MCP-1 | TNF-α |
| --- | --- | --- | --- | --- | --- | --- | --- | --- | --- | --- | --- | --- | --- | --- | --- |
| body mass index | 1 | -0.23 | -0.31 | -0.26 | 0.04 | -0.06 | -0.06 | 0.1 | 0.1 | -0.01 | -0.08 | -0.14 | 0.11 | -0.05 | 0.15 |
| hookworm eggs | 0.000149 | 1 | -0.2 | 0.07 | 0.31 | 0.05 | 0.31 | 0.21 | 0.05 | 0.15 | 0.08 | 0.1 | 0 | -0.01 | -0.02 |
| Hemoglobin | 2.06E-07 | 0.00084 | 1 | 0.21 | -0.29 | -0.01 | -0.13 | -0.28 | -0.03 | -0.07 | -0.01 | -0.07 | 0.05 | 0.07 | 0.11 |
| neutrophils | 8.92E-05 | 0.26 | 0.00104 | 1 | 0.06 | 0.2 | 0.1 | 0.01 | 0.06 | 0.09 | 0.02 | -0.14 | -0.02 | -0.01 | 0.01 |
| lymphocytes | 0.572 | 1.87E-06 | 6.81E-06 | 0.356 | 1 | 0.13 | 0.23 | 0.31 | 0.11 | 0.12 | 0.14 | 0.17 | 0.05 | -0.01 | 0.06 |
| monocytes | 0.369 | 0.469 | 0.905 | 0.00184 | 0.0405 | 1 | -0.01 | -0.02 | 0.14 | 0.14 | 0.05 | -0.01 | 0.1 | 0.03 | 0.13 |
| eosinophils | 0.341 | 1.50E-06 | 0.0561 | 0.11 | 0.000305 | 0.91 | 1 | 0.34 | 0.12 | 0.24 | 0.19 | 0.07 | -0.02 | -0.11 | -0.03 |
| basophils | 0.142 | 0.00125 | 1.21E-05 | 0.907 | 9.95E-07 | 0.74 | 9.21E-08 | 1 | 0.04 | 0.06 | 0.09 | -0.01 | 0.07 | -0.04 | 0.01 |
| GM-CSF | 0.321 | 0.635 | 0.781 | 0.57 | 0.243 | 0.163 | 0.217 | 0.705 | 1 | 0.14 | 0.77 | -0.04 | 0.71 | 0.56 | 0.71 |
| IFNγ | 0.951 | 0.112 | 0.466 | 0.323 | 0.222 | 0.144 | 0.0119 | 0.546 | 0.15 | 1 | 0.11 | 0.18 | -0.03 | -0.08 | 0.01 |
| IL-6 | 0.365 | 0.369 | 0.921 | 0.817 | 0.15 | 0.565 | 0.045 | 0.37 | 1.18E-22 | 0.231 | 1 | 0 | 0.66 | 0.68 | 0.6 |
| IL-10 | 0.153 | 0.274 | 0.449 | 0.134 | 0.0782 | 0.913 | 0.471 | 0.952 | 0.715 | 0.0536 | 0.977 | 1 | -0.04 | -0.06 | -0.02 |
| IL-18 | 0.272 | 0.973 | 0.611 | 0.847 | 0.629 | 0.323 | 0.861 | 0.512 | 2.48E-17 | 0.769 | 4.08E-15 | 0.693 | 1 | 0.66 | 0.73 |
| MCP-1 | 0.621 | 0.877 | 0.44 | 0.881 | 0.88 | 0.743 | 0.264 | 0.65 | 3.57E-10 | 0.387 | 7.08E-17 | 0.518 | 1.57E-14 | 1 | 0.57 |
| TNFα | 0.206 | 0.837 | 0.358 | 0.907 | 0.649 | 0.277 | 0.807 | 0.94 | 2.88E-12 | 0.91 | 2.88E-08 | 0.875 | 4.55E-13 | 1.75E-07 | 1 |

**Supplementary table 2**. Summary of binomial Generalized Linear Mixed Models (GLMMs) evaluating the association between, physiological, immunological and hematological variables and anemia recovery status. Models include animal ID as a random factor and controlled for the effect of age.

| Predictor | Estimate | SE | Z | P |
| --- | --- | --- | --- | --- |
| IFNγ | -0.0002991 | 0.02299835 | -0.0130042 | 0.98962442 |
| IL-6 | -0.0031274 | 0.01499091 | -0.2086216 | 0.83474361 |
| IL-10 | 0.0016131 | 0.01397642 | 0.11541606 | 0.90811535 |
| IL-18 | 0.00084685 | 0.0077091 | 0.1098504 | 0.91252802 |
| MCP-1 | -0.0005665 | 0.00841676 | -0.0673079 | 0.94633661 |
| TNF-α | 0.00057033 | 0.01595464 | 0.03574676 | 0.97148428 |
| GMCSF | 0.00032591 | 0.00682003 | 0.04778776 | 0.96188539 |
| Neutrophils | -4.2E-05 | 0.000345 | -0.12171 | 0.903125 |
| Lymphocytes | -6.743E-05 | 0.00186299 | -0.0361923 | 0.97112902 |
| Basophils | -0.0002952 | 0.00206954 | -0.1426321 | 0.88658076 |
| Monocytes | -0.0002282 | 0.00130288 | -0.1751728 | 0.86094384 |
| Eosinophils | -1.231E-05 | 0.00099056 | -0.0124307 | 0.99008198 |
| Body Mass | 33.9691427 | 105.411746 | 0.32225197 | 0.74726182 |
| Hemoglobin | 0.02391164 | 0.66110939 | 0.03616896 | 0.97114764 |
| Hookworms | 0.01708596 | 0.16743457 | 0.10204557 | 0.9187205 |

**Supplementary table 3**. Comparison of immune and physiological variables between anemia recovered and not-recovered individuals using Wilcoxon rank-sum tests, including adjusted p-values for multiple comparisons (Benjamini–Hochberg procedure).

| **Variable** | **Wilcoxon statistic** | **P value** | **n recovered** | **n not-recovered** | **Adjusted P value** |
| --- | --- | --- | --- | --- | --- |
| Growth Rate | 70 | 1 | 14 | 10 | 1 |
| Hookworm burden | 52.5 | 0.712 | 14 | 10 | 1 |
| Hemoglobin | 81 | 0.14218 | 14 | 10 | 0.297654133 |
| GM-CSF | 30 | 0.01977 | 14 | 10 | 0.059309986 |
| IFNγ | 12 | 0.000274 | 14 | 10 | 0.002057355 |
| IL-6 | 25 | 0.009109 | 14 | 10 | 0.034160147 |
| IL-10 | 120 | 0.002432 | 14 | 10 | 0.012160575 |
| IL-18 | 52.5 | 0.306458 | 14 | 10 | 0.459686981 |
| MCP-1 | 44 | 0.12511 | 14 | 10 | 0.234581994 |
| TNF-α | 46 | 0.070476 | 14 | 10 | 0.176190954 |
| Neutrophils | 9 | 0.00017 | 13 | 10 | 0.002057355 |
| Lymphocytes | 59 | 0.738062 | 13 | 10 | 0.851610363 |
| Macrophages | 44 | 0.208044 | 13 | 10 | 0.346739903 |
| Eosinophils | 51 | 0.409952 | 13 | 10 | 0.512439842 |
| Basophils | 65.5 | 1 | 13 | 10 | 1 |
| Iron | 100 | 0.084128 | 14 | 10 | 0.180273691 |

Supplementary figures

**Supplementary figure 1**. Number of hookworm eggs per smear in 83 South American fur seal pups. Circles represent raw values for one or more pups sampled in a single capture event (date). The number of hookworm eggs increased until mid-January and then gradually decreased until mid-March. By late-February and early-March (end of the study) no pups were found to have hookworm eggs in their fecal smears.
